# A New Criterion for Fluoroquinolone-Associated Disability Diagnosis: Functional Gastrointestinal Disorders

**DOI:** 10.3390/medicina57121371

**Published:** 2021-12-16

**Authors:** Deanna N. Cannizzaro, Lydia F. Naughton, Maya Z. Freeman, Linda Martin, Charles L. Bennett, Cecilia Bove

**Affiliations:** 1 Department of Biology, Bucknell University, Lewisburg, PA 17387, USA; dcanizzaro23@gmail.com (D.N.C.); lfn1671@uncw.edu (L.F.N.); mzf003@bucknell.edu (M.Z.F.); 2 SONAR (Southern Network on Adverse Reactions) Program, College of Pharmacy, University of South Carolina, Columbia, SC 29208, USA; lmartinanthem@cox.net (L.M.); bennetc@cop.sc.edu (C.L.B.)

**Keywords:** fluoroquinolones, Functional Gastrointestinal Disorder, fluoroquinolone associated disability, adverse drug reaction

## Abstract

*Background and Objectives:* Fluoroquinolones (FQs) are a broad-spectrum class of antibiotics routinely prescribed for common bacterial infections despite recent recommendations to use them only for life-threatening cases. In addition to their antimicrobial properties, FQs act in the central nervous system as GABA_A_ receptor inhibitors, which could potentially affect functionality of the vagus nerve at the forefront of gastrointestinal (GI) tract function. Alterations in neural control of digestion have been shown to be linked to Functional Gastrointestinal Disorders (FGIDs), which are usually diagnosed based on self-reported symptoms. The aim of this study was to assess the incidence of FGIDs following FQ use. *Materials and* *Methods*: Self-reports from the FDA Adverse Event Reporting System were analyzed together with ~300 survey responses from a social network derived sample to the Bowel Disease Questionnaire. *Results:* The results of this study suggested that six different FQs are associated with a wide range of GI symptoms not currently reported in the drugs’ labels. The responses from the survey suggested that ~70% of FQ users scored positive for FGID, with no positive correlation between drug type, duration of administration, dosage and frequency of administration. *Conclusions*: This study showed that GI disorders other than nausea, vomiting and diarrhea are more common than currently reported on the drug labels, and that FGIDs are possibly a common consequence of FQ use even after single use.

## 1. Introduction

Fluoroquinolones (FQs) are a class of broad-spectrum antibiotics that received Food and Drug Administration (FDA) approval in 1986 for a wide range of bacterial infections. More than 22 million prescriptions are written yearly for FQs in the United States [1]. FQs act on both Gram-negative and Gram-positive bacteria by inhibiting the type II DNA topoisomerases (gyrases), essential for bacterial mRNA transcription and DNA duplication [2]. While extremely effective, FQs can be accompanied by a variety of systemic side effects, including gastrointestinal disturbances, headaches, skin rash, allergic reactions [3], QT prolongation [3], seizures [4], hallucinations [5], tendon rupture [6,7], etc. While many of the initially identified adverse effects were described as short lasting, more recently, identified side effects are reported by patients as lasting for long periods, potentially decades. With many affected individuals reporting these side effects, in 2015, an epidemiologist from the FDA described a syndrome that she termed as “Fluoroquinolones-associated disability” (FQAD) or “Fluoroquinolones toxicity syndrome” [8].

As operationally defined, FQAD is characterized as occurring in an individual with FQ-associated adverse events involving “two or more” of the following categories: musculoskeletal, neuropsychiatric, peripheral and sensory nervous system, skin, and/or cardiovascular and the adverse events have “to last 30 days or longer after stopping the fluoroquinolone” [9,10]. To date, the term FQAD has not been recognized as a distinct clinical entity by the International Classification of Diseases-10 (ICD-10). Most concerning is the fact that while between 2016 and 2020 “black box label warnings” were added to FQs internationally, clinicians are still not well informed about the toxicities of FQs and international re-assessments of their risk-benefit profiles. In spite of FQ label changes, which indicated in the United States and in Europe that FQs should be reserved for use in patients who have no alternative treatment options for the treatment of uncomplicated urinary tract infection, acute bacterial exacerbation of chronic bronchitis, and acute bacterial sinusitis, FQs continue to be prescribed frequently for routine infections in these regions. A striking 40% of common urinary tract infections has been managed with the use of FQs [11,12]. 

A Functional GI disorder (FGID) is a disorder that disrupts the normal behavior of the GI tract due to motility disturbance, visceral hypersensitivity, altered mucosal and immune function, altered gut microbiota, and altered central nervous system (CNS) processing. This classification includes both Irritable Bowel Syndrome (IBS) and Functional Dyspepsia (FD) [13,14]. Notably, the concept of FGIDs has gained traction relatively recently; indeed, while organic and motility disorders can be tested and validated in the clinical setting, FGIDs rely on patients reporting of the symptoms [13]. Even today, FGIDs are not entirely legitimized compared to organic and motility disorders, even though these three groups of diseases can oftentimes overlap in their presentation. Nonetheless, FGIDs are notably associated with CNS dysregulation at the vagal level [15]. This cranial nerve emerges from the brainstem medulla oblongata at the level of the dorsal motor nucleus of the vagus (DMV) [16,17,18]. DMV neurons tonically regulate gastrointestinal (GI) motility. In turn, DMV neurons are regulated by the neighboring nucleus of the tractus solitarius (NTS), which mainly releases γ-aminobutyric acid (GABA), and secondarily glutamate and other neurotransmitters onto these parasympathetic cholinergic neurons. The tonic GABA-mediated regulation of DMV activity is interrupted on demand by peripheral, afferent vagal signals [19,20,21]. GABA released by the NTS is crucial for the correct functioning of this circuit, referred to as the vago-vagal reflex [19,22,23]. 

Studies on the CNS have shown that the frequently prescribed FQs ciprofloxacin and levofloxacin [24], are selective antagonists of GABA_A_ receptors, which are widely expressed in the CNS [25,26,27]. Previous studies have shown that FQs reduce the GABA-dependent potentials recorded in vitro from vagus nerve preparations in the rat [26,28]. Moreover, the molecular structure of FQs allows for chelation of several cations, including Mg^2+^ [29,30], which ensures the correct gating of *N*-methyl-d-aspartate (NMDA) receptors. These, together with GABA_A_ receptors, are also expressed by DMV neurons. Considering that FQs have been found to potentially create stable chelation complexes with cations [31,32,33,34,35], and considering that the structure of FQs is similar to that of kynurenic acid, an endogenous ligand of NMDA receptors [36], alterations on DMV and, consequently, vagal activity could be occurring on two levels: one by reducing the GABAergic transmission, and two by potentiating glutamatergic transmission through disinhibition and/or agonistic binding to NMDA receptors. In support of this possibility, a study by De Sarro and collaborators showed that pefloxacin can induce seizures in DBA/2 mice with a mechanism involving both GABA and glutamate transmission [36]. Similar findings were published in the genetically absence-prone epilepsy rat model (Wag/Rij) following i.p. Injection of ciprofloxacin [37]. 

In the “Clinical Trial Experience” section of the Levaquin© (levofloxacin; Johnson and Johnson, New Brunswick, NJ, USA) label, common Levaquin© clinical trial adverse events included “nausea, diarrhea, constipation, abdominal pain, vomiting, dyspepsia.” Additionally, the Levaquin© label describes less common GI adverse events including, “gastritis, stomatitis, pancreatitis, esophagitis, gastroenteritis, glossitis, pseudomembranous/C. difficile colitis”. The label for Cipro© (ciprofloxacin; Bayer, Leverkusen, Germany) is similarly structured [38]. Furthermore, the “Postmarketing Reports of Adverse Drug Reactions’’ section of the Levaquin label does not report instances of GI adverse effects [39]. However, considering the difficulty in diagnosing FGIDs, it is possible that GI disturbances may be part of the clinical picture of FQAD.

The purpose of this study is to evaluate the range of adverse GI drug reactions associated with utilization of Fluoroquinolones, in particular FGIDs, to determine whether GI adverse effects should be considered as part of the clinical picture in FQAD.

## 2. Materials and Methods

### 2.1. FDA Adverse Event Reporting System Data Collection

The FDA Adverse Event Reporting System (FAERS) Dashboard was used to identify human adverse events reported to the FDA. FAERS is a database used in the United States to collect post-marketing safety data on approved drugs and therapeutic products, and it is accessible to drug consumers, health care professionals and drug manufacturers. While the latter are required to submit safety reports to the FDA, health care professionals and consumers can choose to do so voluntarily. 

For this study, queries included: (1) Cipro©/ciprofloxacin, (2) Levaquin©/levofloxacin, (3) Avelox©/moxifloxacin, (4) Norfloxacin, (5) Lomefloxacin, and (6) Ofloxacin. For each drug, “Gastrointestinal Disorders” were selected in the “Reaction Group” Table The data discussed in this study reflect claims collected between 2004 and March 2021.

### 2.2. Study Participants

The survey was distributed to individuals who participated in FQ-related social media communities on different social media platforms including Twitter, Facebook groups such as “Fluoroquinolone Toxicity 24/7 Live Chat Group”, “Fluoroquinolone Antibiotic Toxicity Wall of pain UK” and “Fluorochinoloni—Gruppo di Supporto per i Danneggiati da Antibiotico” (Italy, nationwide group), and Reddit on the forum “r/floxies”. Data were collected from July 2020 to June 2021. Potential study participants were informed about the project and recruited online. Reminders were sent over the whole study period (11 months). In total, 370 FQ users from different countries (mainly the United States, Italy, and United Kingdom) agreed to participate in this study with an overall response rate of 90.5%. Inclusion criteria included age (participants were at least 18-years old) and previous utilization of FQs. Participants were excluded from the study if they presented with a history of prior GI disorders, as assessed through the questionnaire. Based on the responses received, the data were divided into 4 main groups: (1) ciprofloxacin, (2) levofloxacin, (3) other FQs (including lomefloxacin, moxifloxacin, norfloxacin, ofloxacin), and (4) combination, when a patient took more than one type of FQ in their lifetime. 

### 2.3. The Bowel Disease Questionnaire

For this study, a modified version of the Bowel Disease Questionnaire by Talley et al. [40] was used. Briefly, the questionnaire addresses 46 GI symptoms, 16 past and present health items, 1 childhood question, 3 sociodemographic items, 5 health habit questions, and 17 questions adapted from the Psychosomatic Symptom Checklist, plus 4 questions regarding FQs that we integrated into the original questionnaire. The diagnostic validity of the questionnaire was verified in the original study by means of logistic discriminant analysis to examine the diagnostic validity of the a priori scores used for diagnosis (see Data analysis) and further validated with a cross-validation approach. The reliability of the original questionnaire was evaluated with the kappa statistics [40].

In addition to the original survey questions, participants were asked what kind of FQs they have been administered, the length of their treatment, the dosage, and frequency of utilization per day.

### 2.4. Data Analysis

Pearson chi-square tests for independence (categorical variables) was used to examine the reported adverse drug reactions extrapolated from the FAERS database. Degrees of freedom for the Pearson chi-square test were defined as follows: (r − 1) × (c − 1), where r = the number of rows and c = the number of columns. Significance level was set at *p* = 0.05.

For portions of the questionnaire that required an open-ended answer (i.e., length of the FQs treatment and dosage), if a range was provided, an average data point was calculated. In the case of individuals that went through multiple antibiotic courses, only the highest reported value was included in the analysis.

The answers to the GI portion of the survey were analyzed based on four a priori scores following the methodology included in the original manuscript by Talley and collaborators [40]. Briefly, four a priori scores were calculated based on answers to questions selected to describe symptoms associated with IBS, FD, Bowel Habits, and Pain. These scores were then used to classify each patient as likely to have (1) FGIDs, (2) another GI disease, and (3) no GI disease (healthy).

The relationship between the patients’ diagnostic scores and dosage, length of treatment, or frequency of treatment with the various FQs examined was performed by calculating the Pearson R^2^ coefficient (significance level: 0.05). All data analyses were performed in XLMiner Analysis Pro.

## 3. Results

### 3.1. FAERS Reports on Gastrointestinal Disorders

Between 2004 and March 2021, 45,485 cases of adverse events were reported to the FAERS for Cipro©/ciprofloxacin, the vast majority including serious cases; a similar trend was observed for Levaquin©/levofloxacin, lomefloxacin, Avelox©/moxifloxacin (Bayer, Leverkusen, Germany), norfloxacin, and ofloxacin (49,116; 1500; 20,705; 3022 and 10,003 total cases respectively). Analysis of the adverse reactions revealed that GI cases were reported with each of the FQs analyzed; specifically, 20% of ciprofloxacin and moxifloxacin users reported GI adverse side effects, followed by 17.86% for norfloxacin, 15.89% for ofloxacin, 14.86% for lomefloxacin, and 14.82% for levofloxacin. Analysis of the relationship between the type of FQ and the reported side effects indicated that the number of ADRs defined as serious is greater for levofloxacin than the expected value; death appears to be observed more than expected with ofloxacin and norfloxacin; and the total number of reported gastrointestinal ADRs is greater than expected for ciprofloxacin, moxifloxacin, and lomefloxacin (*p* < 0.05). Data are summarized in Table 1.

Notably, the vast majority of the GI cases associated with FQs were considered “Serious cases”. Based on the total number of ADRs reported, the data shows that serious GI cases have an incidence rate of 10/1000 cases per year for ciprofloxacin, 8/1000 for levofloxacin, 9/1000 for moxifloxacin and norfloxacin, and 5/1000 for lomefloxacin. Furthermore, 12.98% reported being permanently disabled by ciprofloxacin, 10.68% by levofloxacin, 9.44% by norfloxacin, 4.59% by ofloxacin, 4.39% by moxifloxacin, and 0.45% by lomefloxacin. Reports of death were found for each of the FQs. Ciprofloxacin, levofloxacin, lomefloxacin and moxifloxacin seem to be associated with serious GI side effects more than expected; similarly, the number of disabling GI side effects are observed more frequently than expected with ciprofloxacin, levofloxacin and norfloxacin (*p* < 0.05). Data are summarized in Table 2.

**Figure 1:**
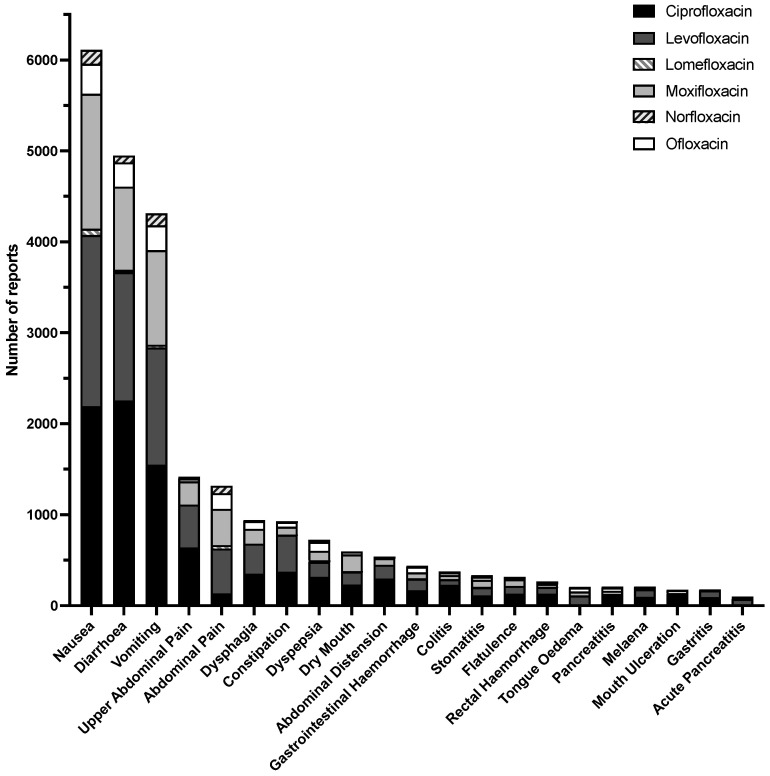
Gastrointestinal adverse events reported between 2004 and March 2021 in the FAERS database for ciprofloxacin (black), levofloxacin (dark grey), lomefloxacin (striped light gray), moxifloxacin (light gray), norfloxacin (striped dark gray), and ofloxacin (white).

Further examination of specific GI symptoms revealed that some symptoms were reported with all FQs analyzed (i.e., nausea, diarrhea, vomiting, etc.), while some were only experienced by 5 or 4 of the FQs (i.e., mouth ulceration, pancreatitis, etc.). Crohn’s Disease and IBS appeared to be in the top 30 reported adverse events only for ciprofloxacin. A summary of the top adverse GI side events reported can be found in Figure 1.

These data suggest that GI disorders other than nausea, diarrhea and vomiting should be included in product labels for all FQs, and that serious side effects should be considered uncommon side effects.

### 3.2. Survey Responses

The FAERS data did not include information on the duration of GI symptoms. Hence, a social media-based survey was administered to a cohort of individuals who had used FQs asking detailed questions on their GI health in the last 12 months. Of the 370 participants recruited, 33 were excluded from the study due to pre-existing GI conditions and 31 withdrew from the study or did not complete it. In total, 306 responses were collected. Of these respondents, 154 reported taking ciprofloxacin, 69 levofloxacin, 21 other FQs, 61 a combination of two or more FQs, and 1 participant was not able to answer. This patient was not scored for diagnostic purposes and was not included in the correlation analysis. The data describing the therapeutic regimen with FQs are summarized in Table 3.

#### 3.2.1. Upper and Lower Abdominal Issues

Approximately 80% of the respondents reported stomach pain/discomfort in the last year. Of this fraction, 86% experienced pain more than 6 times in the last year, which ~49% considered to be severe/very severe. The pain was equally localized above or below the navel, with 47% of the patients reporting the pain migrating between the two locations. The majority of the patients indicated that this pain would come and go periodically (65%), with 51.5% of individuals reporting that the pain occurred on a daily basis or several times a week. The majority of patients indicated that the pain would last between 30–120 min (36%), while others experienced this pain for more than two, up to six hours (24 and 26% respectively). About half of the participants experienced this pain immediately after meals, or 30–120 min following a meal. In addition, 57% of people reported the pain being worsened by food or milk. However, this pain was not alleviated after bowel movements for half of these individuals, nor after eating (81%). Finally, 47% of the respondents reported having more bowel movements at the start of the painful sensation, with 58% reporting loose stools.

The data follow a similar trend when broken down by drug type. Of note, in participants who only took ciprofloxacin, 84% reported having pain more than six times in the last year; 39% of these reported that the pain was moderate, and 36% reported having this pain multiple times a week. 

With levofloxacin, 41% of participants reported having pain multiple times a week, with 33% experiencing this pain between 30–120 min. A marked 85% of respondents reported that the pain is not ameliorated with food; in 51% of the reports, food and milk worsen the pain. Sixty percent of respondents reported looser bowel movements when the pain starts. 

Patients who took other types of FQs rated their pain as moderate (54%), lasting 30–120 min (46%), generally as digestion starts (30–120 min after a meal; 76%). A similar percentage of participants reported not feeling better after eating. Sixty-nine percent of respondents report having more bowel movements with loose stools after the pain starts.

Lastly, we analyzed the responses of patients who took a combination of two or more FQs in their lifetime. Almost half of the respondents (45%) rated the pain as severe, with 62% reporting disrupted sleep because of the pain, several times a week (27%), lasting 30–120 min (31%), most of the time after a meal (74%). Similar to what was observed with other FQs, the pain was not alleviated by food consumption (80%), nor after having a bowel movement (52%), which were more frequent and looser (51% and 66%, respectively). 

#### 3.2.2. Bowel Habits

During the twelve months preceding the survey, 50% of the respondents reported changes in their usual bowel habits. Specifically, 16% of this fraction reported being constipated or experiencing diarrhea, and 35% reported an alternation between constipation and diarrhea. Nonetheless, 39% of respondents reported an average of 5–8 bowel movements per week, 21% reported 9–12 bowel movements per week, and fewer respondents reported either severe constipation (four bowel movements or less) or diarrhea (between 13–16 bowel movements or more frequent). Few participants indicated often having less than three (11%) or more than three (23%) bowel movements. Forty-five percent of survey respondents indicated the presence of mucus in their stools. Interestingly, 62% of respondents reported feeling as if their bowel movements were incomplete more than 25% of the time, with 48% indicating that they often have an urgent need to empty their bowels. Strikingly, 87% of the respondents stated observing blood on the toilet paper after emptying their bowels. 

Of those who only took ciprofloxacin, 59% reported that they frequently feel stool to be passed after having a bowel movement, and a striking 91% reported blood on the toilet paper. 

Levofloxacin-treated respondents reported bowel habits alternating between diarrhea and constipation (36%), with 51% of respondents reporting often straining to have bowel movements, with hard stools (55%) and feeling of incomplete bowel emptying (65%). Similar to what described above, 79% reported seeing blood on the toilet paper.

In the group of patients that consumed FQs other than ciprofloxacin and levofloxacin, 67% reported feeling of incomplete evacuation, with 52% reporting they often strain to pass stools. All of the respondents reported the presence of blood on the toilet paper. 

Lastly, for those who took a combination of FQs, 59% reported a change in bowel habits, with alternation between constipation and diarrhea (39%). Unsurprisingly, 62% reported having loose or watery stools, with a feeling of incomplete evacuation and 55% reported having an often and urgent need to pass a bowel movement. Ninety percent of respondents reported seeing blood on the toilet paper.

#### 3.2.3. Nausea, Appetite, and Heartburn

Although the majority (78%) of patients reported feeling nauseated in varying degrees of frequency, few experienced vomiting in the last year (39%). Seventy-one percent of individuals often felt bloated and saw abdominal swelling, with 46% reporting difficulty swallowing and 77% reporting experiencing heartburn sometimes accompanied by reflux (62%). Lastly, some patients reported unexplained weight loss or gain (17 and 26% respectively). Almost half of the respondents (42%) reported reduced appetite.

Bloating appeared to be frequent in patients treated with ciprofloxacin (68%) and levofloxacin (75%). Other FQs seem to not induce swelling to the same magnitude (75%), while a history of multiple treatments with different FQs seem to have the worst rate of bloating cases (82%), with 57% of patients also reporting dysphagia and a similar trend in appetite decrease compared to the general pool of respondents. 

Overall, these data suggest that FQ-associated GI disorders may occur frequently and that these symptoms persist even after the cessation of FQ treatment for longer than 30 days.

#### 3.2.4. Patient Scoring

Next, we utilized the criteria described by Talley and collaborators [40] to score patients based on GI symptoms reported. Seventy percent of the survey respondents reported symptoms consistent with FGID, 13% with other GI disorders, and only 17% can be considered healthy. Specifically, 76% of levofloxacin users meet criteria for FGID, followed by 72% of ciprofloxacin users, and 62% of other FQ users. Interestingly, a history of using more than one FQ during one’s lifetime does not increase the possibility of developing FGID (59%).

In order to determine whether or not factors such as dosage of the drug, duration of the treatment, or frequency of administration impacted the scoring findings, a correlation analysis was conducted for each of the groups analyzed. The a priori scores for Irritable Bowel Syndrome, Functional Dyspepsia, Bowel Habits, and Pain used to classify patients as likely to have FGID do not show to be correlated with the drug dosage, duration of treatment, nor the frequency of antibiotic administration for ciprofloxacin and levofloxacin. A weak positive correlation was found for the Functional Dyspepsia score versus the duration of the treatment, the Bowel Habits score versus dosage, and the Pain score versus duration of the treatment with other FQs. Data are summarized in Table 4.

These data indicate that administration of even one dose of any of the FQs analyzed was associated with long-term FGID in otherwise healthy individuals.

## 4. Discussion

This study presented data supporting adding FGIDs as a toxicity to labels for FQs. Specifically, (1) the range of GI adverse events reported both by survey respondents and in the FAERS database includes a variety of specific symptoms, such as dyspepsia, dysphagia, pancreatitis (acute and not), constipation, gastritis, colitis, bloating and persistent abdominal pain that are not accurately captured in the labels, (2) the symptoms reported in the survey are in line with the diagnostic criteria for FGIDs, and (3) GI symptoms persisted for more than 30 days and were experienced by persons who had previously reported no GI symptoms.

It is common knowledge that antibiotics are associated with temporary, mild gastrointestinal symptoms. Antibiotic-associated diarrhea, amongst other symptoms, is the most common adverse effect due to a disruption of the host microbiome, which tends to resolve on its own as soon as the therapy is discontinued [41]. Indeed, the labels for all FQs analyzed in this study alert the consumer about common, mild gastrointestinal adverse events [38,39,42,43,44,45], as well as about some uncommon, severe GI disorders. However, based on the findings of this study, we believe that the labels do not currently describe the possibility of severe GI side effects lasting for longer than 30 days, a time frame that would align with the definition of FQAD [9,10].

Our analysis of the FAERS data clearly shows that while nausea, vomiting, and diarrhea are still the top three side effects reported with each of the six FQs analyzed, other symptoms are frequently reported. Of particular importance is the notion that more than 70% of the gastrointestinal symptoms reported were described as severe, while current labels for FQs state that GI symptoms are generally mild and of short duration. A limitation to this analysis is that it is not possible to determine the duration of these adverse events in a population of FQ users included in FAERS; hence the need of the GI questionnaire in a population-based survey. As designed, the questionnaire does not address when the respondent underwent therapy with FQs; however, it specifically required the respondents to report GI symptoms in the last 12 months. By reducing the possibility of surveying FQ users currently on treatment, the likelihood of including temporary, mild GI side effects in our data were eliminated. It is important to notice that the vast majority of the respondents clearly stated that they were not currently treated with FQs. Moreover, survey participants with pre-existing GI conditions and/or major GI surgeries were excluded from the study. As such, the symptoms described are most likely long-lasting (more than 30 days from the end of the therapy with FQs) and could possibly be caused by therapy with FQs. In light of our results, we propose to revisit the current labels for FQs to include the symptoms reported in the FAERS database and to expand FQ product labels to include instances of severe and long-lasting GI disorders, in particular FGIDs.

The most surprising and significant evidence reported in this study is the lack of a correlation between drug therapy and the symptoms reported. Indeed, dosage, duration of the treatment, and the per diem frequency of administration did not affect the chance of experiencing severe and long-lasting GI distress. This is especially relevant when considering that a significant portion of the respondents appears to have a high likelihood of being affected by a FGID. In light of our findings, FQs administration should be avoided especially in subjects with pre-existing GI complications. This is particularly important considering that a meta-analysis review supports the use of ciprofloxacin and norfloxacin in treating traveler’s diarrhea as prophylaxis for irritable bowel syndrome [46], a common FGID. Based on our results, we discourage these antibiotics for prophylaxis.

FGIDs are best defined by their symptoms [40,47,48,49,50,51,52,53,54] and as such have been difficult to diagnose, even after 30 years of research on the topic [13]. As such, FGIDs were considered by most illegitimate and stigmatized as unreal, or psychosomatic in nature [13,55]. While today we have better criteria for the assessment of FGIDs [56] and a clear definition of this group of diseases by the Rome IV committee [13], disease management and diagnosis are still complex due to the multifaceted nature of FGIDs. The Rome IV criteria as they stand are useful for pharmaceutical and clinical trials but pose limitations in clinical practice. The most challenging aspect of FGIDs diagnosis is tied to the psychosocial factors feeding into the presentation of the symptoms: psychological stress, a history of trauma, and maladaptive cognitions greatly affect the clinical outcome. Of particular importance is the notion that GI disorders in and of themselves impact an individual’s wellbeing and can have disruptive consequences on one’s mood and ability to function daily. As such, a vicious cycle where mood disorders and FGIDs symptoms feed into each other can arise [13]. While this notion alone is already a cause of concern, it is even more relevant when putting FGIDs in the context of FQAD. FQ use is notoriously associated with mood disturbances, including anxiety, depression and even suicidal ideation [57,58,59,60]. With this in mind, the use of FQs should be advised against, or at the very least, FGIDs should be mentioned in the labels for these drugs. At the same time, gastroenterologists should be on the lookout for prior FQ use as a possible risk factor for FGIDs onset when determining diagnosis and disease management strategies.

While some mechanisms of cellular toxicity have been identified for FQs, including metal cations deposition [29,31,34,61,62,63], mitochondrial damage and increase in oxidative stress [30,64,65,66,67,68,69], it is still unclear whether or not a single exposure to FQs is sufficient to trigger these pathophysiological mechanisms, or if repeated exposure leads to cellular, tissue and then organ dysfunction. This is the reason why it was imperative to administer the survey on individuals that went through multiple courses of the same FQ or to different FQs. The rate of FGIDs seems to be lower, although only marginally, in individuals that were exposed to more than one kind of FQ. As such, we cannot exclude the possibility that one single dose of any of the FQs examined herein could be sufficient to increase the odds of developing a FGID in individuals without pre-existing GI conditions. While this study is purely observational and lacks the advantages of a close clinical examination of the subjects enrolled in the study, some speculations can be made regarding the pathophysiological mechanisms of FGIDs in FQAD. 

One possibility is that FQs exert harmful effects on the host microbiome. It is well established that disturbances in the microbiome affect the gut-brain axis and can induce FGIDs [70,71]. A previous study showed that ciprofloxacin can reduce the number of enterobacteria in cultured fecal samples [72]. A more in-depth analysis of 16S rRNA isolated from the fecal microbiome of patients treated with the same drug showed a marked change in microbiota composition, with only partial recovery of the microbiota composition after discontinuation of the treatment [73]. In contrast to these findings, another study showed little changes in the microbiota in hospitalized patients [74]. While these results can point to a disruption of the physiological composition of the microbiota to explain the onset of FGIDs, it is important to keep in mind that the vagus nerve is at the center of the brain-gut axis: indeed, changes in microbiome composition can affect the functionality of this cranial nerve [70]. The vagus nerve has been severely understudied in the context of FQ toxicity; however, some animal-based research studies have confirmed a specific antagonism of FQs against GABA_A_ receptors on the vagus nerve [28] that were not observed in nerve preparations of the optic nerve [26], potentially suggesting that the vagus might be particularly vulnerable to FQs toxicity. Considering the modest liposolubility of FQs [75], the possibility that FQs may induce FGIDs through a combination of direct impairment of the vagus nerve and disruption of the host microbiome composition should not be discounted.

The results of this study are limited by the fact that it relied on self-reports. First, there is no certainty that the reported events were due to the product. Reports do not always contain enough detail to properly evaluate these events. There is also the possibility of duplicate reports where the same adverse event was submitted by a consumer and by the manufacturer, the former being on a voluntary basis and the latter being required by the FDA. The lack of measurable parameters to assess the GI tract due to the methodological constraints of this study poses clear limitations. However, the questionnaire we based our investigation on has been validated as a diagnostic tool for FGIDs [40]. A retrospective study should be conducted to confirm whether or not there is a relationship between FQs and the onset of severe gastrointestinal complications, and to compare the incidence of FGIDs with other commonly prescribed antibiotics. Furthermore, additional research is necessary to evaluate the relationship between FQs, the vagus nerve, and the gut microbiome in a reliable animal model in order to propose a pathophysiological mechanism able to fully explain our results.

## 5. Conclusions

In conclusion, our findings suggest another toxicity should be discussed with patients who are prescribed FQs. Additionally, this study concludes that FGIDs should be included in the constellation of FQ adverse events that may be long lasting. Gastroenterologists should be aware of the relationship between FQs and FGIDs when evaluating the personal history of their patients, and healthcare providers should become more aware of the dangers of FQAD. It is imperative to discuss this possibility with individuals who are prescribed FQs, and to comprehensively study the topic of long-term toxicity associated with FQs to correctly diagnose, legitimize, and provide disease management approaches for FQAD. Overall, antibiotic stewardship for future cohorts of patients is essential, as the risk–benefit profile of FQs has changed dramatically since 2016.

## Figures and Tables

**Table 1:** Summary of the total number of adverse reactions, serious cases, death cases, and total gastrointestinal (GI) cases for the six FQs analyzed in the present study. Bolded values indicate the possibility of a relationship between a drug and an adverse drug reaction as analyzed through chi-square analysis. CPX, ciprofloxacin; LVX, levofloxacin; MFX, moxifloxacin; NFX, norfloxacin; OFX, ofloxacin; LMX, lomefloxacin.

Drug	Total Cases	Total Serious Cases	Total Death Cases	Total Gastro-Intestinal Cases
Cipro/CPX	45,485	36,993 (81.33%)	3193 (7.02%)	**9098 (20.00%)**
Levaquin/LVX	49,116	**41,470 (84.43%)**	3124 (6.36%)	7283 (14.82%)
Avelox/MFX	20,705	14,558 (70.31%)	1252 (6.05%)	**4347 (20%)**
OFX	10,003	7695 (76.93%)	**1493 (14.92%)**	1590 (15.89%)
NFX	3022	2526 (83.59%)	**331 (10.95%)**	540 (17.86%)
LMX	1500	1270 (84.67%)	12 (0.8%)	**223 (14.86%)**

**Table 2:** Summary of the total number of gastrointestinal defined as serious, disabling, or associated with death. Bolded values indicate the possibility of a relationship between a drug and an adverse drug reaction as analyzed through chi-square analysis. CPX, ciprofloxacin; GI, gastrointestinal; LVX, levofloxacin; MFX, moxifloxacin; NFX, norfloxacin; OFX, ofloxacin; LMX, lomefloxacin.

Drug	GI Total Cases	GI Serious Cases	GI Disabled Cases	GI Death Cases
Cipro/CPX	9098	**7943 (87.30%)**	**1181 (12.98%)**	649 (7.13%)
Levaquin/LVX	7283	**6438 (88.40%)**	**778 (10.68%)**	569 (7.81%)
Avelox/MFX	4347	**3018 (69.43%)**	**191 (4.39%)**	243 (5.59%)
OFX	1590	1192 (74.97%)	73 (4.59%)	214 (13.46%)
NFX	540	442 (81.85%)	51 (9.44%)	33 (6.11%)
LMX	223	**188 (84.30%)**	1 (0.45%)	2 (0.9%)

**Table 3:** Summary of the data collected from all survey respondents regarding the duration of their FQ treatment, dosage, and frequency (per diem) of administration.

Duration of Treatment		Dosage (mg)		Frequency (per Diem)	
Response	n	Response	n	Response	n
Taken once only	17	50	2	Once	85
2–5 days	70	100	1	Twice	171
5–7 days	42	Between 200 and 300	16	3×	20
>7 days	101	Between 300 and 400	16	4×	4
>14 days	33	500	153	Unknown	26
>21 days	12	>500	22		
>1 month	22	Unknown	94		
Unknown	9	FQ not in pill form	2		

**Table 4:** Summary of the R^2^ correlation values for linear regressions calculated to compare the relationship between the diagnostic a priori scores and dosage, duration of treatment, and frequency per diem with ciprofloxacin (n = 154), levofloxacin (n = 69), other FQs (n = 21) and a combination of two or more FQs (n = 61). Bolded values indicate a slight, positive correlation between the two values analyzed; *p* < 0.05.

	Irritable Bowel Syndrome		Functional Dyspepsia		Bowel Habits		Pain	
	Drug	R^2^ Value	Drug	R^2^ Value	Drug	R^2^ Value	Drug	R^2^ Value
Dosage	CPX	0.00002	CPX	0.0008	CPX	0.0006	CPX	0.008
	LVX	0.03	LVX	0.03	LVX	0.01	LVX	0.03
	Other	0	Other	0.04	Other	**0.27**	Other	0.06
	Combo	0.016	Combo	0.005	Combo	0.006	Combo	0.001
Duration of treatment	CPX	0.02	CPX	0.07	CPX	0.04	CPX	0.04
	LVX	0.05	LVX	0.11	LVX	0.04	LVX	0.1
	Other	0.07	Other	**0.28**	Other	0.14	Other	**0.28**
	Combo	0.006	Combo	0.003	Combo	0.01	Combo	0.0003
Frequency per diem	CPX	0.001	CPX	0.0001	CPX	0.006	CPX	0.0003
	LVX	0.02	LVX	0.04	LVX	0.01	LVX	0.03
	Other	0.006	Other	0.17	Other	0.04	Other	0.15
	Combo	0.002	Combo	0.001	Combo	0.006	Combo	0.01

## Data Availability

Data and study material will be made available to other researchers upon request.
